# Rumination Syndrome in Children and Adolescents: A Mini Review

**DOI:** 10.3389/fped.2021.709326

**Published:** 2021-08-19

**Authors:** Marc Martinez, Sandeep Rathod, Hunter J. Friesen, John M. Rosen, Craig A. Friesen, Jennifer V. Schurman

**Affiliations:** ^1^Kansas City University of Medicine and Biosciences, Kansas City, MO, United States; ^2^University of Kansas School of Medicine, Kansas City, MO, United States; ^3^Division of Gastroenteology, Hepatology, and Nutrition, Children's Mercy Kansas City, Kansas City, MO, United States; ^4^Department of Pediatrics, University of Missouri Kansas City School of Medicine, Kansas City, MO, United States

**Keywords:** rumination syndrome, regurgitation, vomiting, gastroesophageal reflux, functional gastrointestinal disorders, diaphragmatic breathing, motility

## Abstract

**Introduction:** Rumination syndrome involves recurrent regurgitation of food and is believed to be underdiagnosed with patients experiencing long delays in diagnosis. It can be associated with significant social consequences, high rates of school absenteeism, and medical complications such as weight loss. The primary aims of the current review are to assess the literature regarding prevalence, pathophysiology, and treatment outcomes with a focus on neurotypical children and adolescents.

**Results:** Population studies in children/adolescents, 5 years of age or older, range from 0 to 5.1%. There are fewer studies in clinical settings, but the prevalence appears to be higher in patients with other gastrointestinal symptoms, particularly chronic vomiting. While physiologic changes that occur during a rumination episode are well-described, the underlying cause is less well-defined. In general, rumination appears to have similarities to other functional gastrointestinal disorders including dysmotility, possibly inflammation, and an interaction with psychologic function. While diaphragmatic breathing is considered the mainstay of treatment, pediatric data demonstrating efficacy is lacking, especially as an isolated treatment.

**Conclusion:** Pediatric rumination syndrome remains greatly understudied, particularly regarding treatment. There is a need to better define prevalence in both the primary care and subspecialty clinical settings, especially in patients presenting with vomiting or apparent gastroesophageal reflux. There is a need to determine whether treatment of co-morbid conditions results in improvement of rumination. Diaphragmatic breathing needs to be studied and compared to other competing responses.

## Introduction

Rumination syndrome in children and adolescents is defined by Rome IV criteria as repeated regurgitation with rechewing or expulsion of food that begins shortly after meal ingestion, does not occur at night, and is not preceded by retching (1). While rumination can occur in any youth, three different groups have been the focus of research: infants/toddlers, neurodiverse youth (e.g., those with psychiatric, neurologic, or developmental disorders), and neurotypical youth (2, 3). Etiologies and treatments vary significantly by group. The focus of the current article is neurotypical children and adolescents.

Rumination is believed to be underdiagnosed, with patients seeing numerous providers and experiencing long delays before diagnosis (4, 5). Patients are frequently believed to have refractory gastro-esophageal reflux (6). Rumination also is frequently associated with other gastrointestinal symptoms—including abdominal pain, nausea, constipation, and diarrhea—which may become the focus of evaluation (5, 7). While the diagnosis of rumination does not require testing, evaluations such as endoscopy, gastric emptying studies, and pH studies are frequently undertaken to evaluate regurgitation (5, 8). While abnormal tests are not uncommon, it is unknown whether abnormal tests are clinically important or identify factors important to generation of rumination. In addition, rumination itself may result in abnormal test results such as an abnormal pH or impedance study generated by frequent rumination.

Rumination is generally believed to be “benign” from the standpoint of physical complications. However, rumination can be associated with significant social consequences, including high rates of school absenteeism, and medical complications have been reported, including dental erosions (5, 7, 9). Rumination syndrome in youth also has been associated with weight loss in 17–43% (5, 7).

This article represents a review of the most current information on rumination syndrome in neurotypical youth (those without co-morbid neurologic or developmental disorders), particularly prevalence, pathophysiology, and treatment outcomes.

### Search Strategy and Selection Criteria

With the assistance of a medical librarian from the Children's Mercy Kansas City, we conducted a systematic search of the literature. Following the PRISMA checklist, we queried five electronic databases: PubMed, Embase, CINAHL, Cochrane CENTRAL, and Scopus from January 1, 2000 to June 30, 2020. Our search strategy centered on Medical Subject Headings (MeSH) and key words relevant to our research question. Key words included: rumination, rumination syndrome, infant, child, adolescent, and pediatric. Additionally, we cross-referenced bibliographies from relevant background articles with our search results to ensure completeness.

We included all studies published from January 1, 2000 through June 30, 2020 that were available in English. Our study population consisted of patients aged 1–21 years of age inclusive. Single case reports were excluded. Series reporting treatment outcome were included only for sample sizes >5. Articles not reporting prevalence, pathophysiology, or treatment outcomes were not included. Letters to the editor, review articles, book chapters, and published abstracts were excluded with the exception that bibliographies from review articles were assessed for additional references. Duplicates were removed by reviewing and comparing abstracts. Remaining titles and abstracts (*N* = 325) were reviewed independently by the authors for eligibility, yielding 49 articles for full review. Data extractions were performed independently by two reviewers (MM and SR). After comparing results, any discrepancies were resolved. Our search yielded 18 manuscripts related to prevalence, 8 related to pathophysiology, and 5 related to treatment in pediatric rumination syndrome.

### Prevalence

Most prevalence estimates have resulted from general population studies, often performed in schools [(10–27); see Table 1]. Estimates are available from countries in North America, South America, Europe, and Asia. Overall, prevalence ranges from 0 to 9.7% with a tendency to be higher in children 4 years of age or younger when studies assess across wide pediatric age ranges. The prevalence in youth 5 years of age or older range from 0 to 5.1%. In these older children and adolescents, the highest prevalence has been reported in two studies in Sri Lanka and one from the United States (18, 23, 27). Outside Sri Lanka, other studies in Asian countries report low prevalence, as do studies of older children in South America (14, 19, 21, 22, 25). All but two of the 18 prevalence studies have utilized the Questionnaire on Pediatric Gastrointestinal Symptoms (QPGS; usually the Rome III version) or the closely related Rome IV Diagnostic Questionnaire. Most often, a translated version of the QPGS was utilized although it is often unclear whether the translated version had been validated. Most studies evaluated prevalence across all pediatric functional gastrointestinal disorders, including reporting of rumination syndrome prevalence data under this broader umbrella. Two studies utilized the Eating Disturbances in Youth Questionnaire (EDY-Q) and assessed specifically for rumination and pica (16, 20). These two studies generally tended to report higher prevalence. Only one study utilized Rome IV criteria, also reporting prevalence by Rome III criteria (21). As compared to Rome III criteria, Rome IV eliminated the requirement that rumination be painless and also unresponsive to treatment directed at gastroesophageal reflux. In this study, rumination prevalence was very similar between the two criteria (0.5 vs. 0.4%) (21).

**Table 1:** Summary of studies reporting prevalence in pediatric rumination syndrome.

**First author**	**Country**	**Population assessed**	**Age range**	**Total population size**	**Diagnostic method**	**Prevalence**
Scarpato et al. (10)	Croatia, Greece, Israel, Italy, Jordan, Lebanon, Macedonia, Serbia, and Spain	General school population	4–18 years	13,750	Rome III questionnaire (translated QPGS); parent report in 4–10 y.o. and self-report in 11–18 y.o.	0.03% in 4–10 year-olds and 0.1% in 11–18 year-olds
Robin et al. (11)	United States	General population	0–18 years	1,255	Rome IV Pediatric diagnostic Questionnaire; parent report	1.7% in infants, 2.1% in toddlers and 0% in 4+ year-olds
Steutel et al. (12)	Belgium, Italy, and The Netherlands	General pediatric or well-baby clinic population	0–48 months	2,751	Rome III questionnaire (QPGS); parent report	4.3% in 0–12 month-olds and 0% in 13–48 month-olds
Altamini et al. (13)	Jordan	General school population	4–18 years	1,587	Rome III questionnaire (QPGS Arabic version); self-report	0.4% in 4–10 year-olds and 0.3% in 11–18 year-olds
Peralta-Palmezano et al. (14)	Colombia	General school population	8–17 years	864	Rome III questionnaire (QPGS Spanish version); self-report	0.5% RS only, 1.3% RS and one other FGID,.1% RS and 2 or 3 other FGID
Bouzios et al. (15)	Greece	General school population	6–18 years	1,588	Rome III questionnaire (QPGS Greek version); parent report in 6–11 y.o. and self-report in 12–18 y.o.	0.5%
Murray et al. (16)	United States, Germany, and Switzerland	General school population	7–13 years	1,430	Eating Disturbances in Youth questionnaire (EDY-Q); self-report	9.7%
Chogle et al. (17)	Colombia	Primary careclinic population	0–4 years	1,231	Rome III questionnaire (QPGS Spanish version); parent report	7.2% in 1–12 month-olds, 2.7% in 13–48 month-olds
Devanarayana et al. (18)	Sri Lanka	General population	12–16 years	427	Rome III questionnaire (QPGS Sinhalese version); self-report	4%
Bhatia et al. (19)	India	General school population	10–17 years	1,115	Rome III questionnaire (QPGS Hindi version); self-report	0.3%
Hartmann et al. (20)	Germany	General population	7–14 years	804	Eating Disturbances in Youth questionnaire (EDY-Q); self-report	11.94% with rumination behavior at least once, 1.49% with recurrent
Saps et al. (21)	Columbia	General school population	8–18 years	3,567	Rome III and IV questionnaires (QPGS Spanish version); self-report	0.5% per Rome IV and 0.4% per Rome III
Sagawa et al. (22)	Japan	General school population	10–17 years	3,976	Rome III questionnaire (QPGS Japanese version); self-report	0.1%
Rajindrajith et al. (23)	Sri Lanka	General school population	10–16 years	2,163	Rome III questionnaire (QPGS Sinhala version); self-report	5.1%
Lewis et al. (24)	United States	General population	4–18 years	949	Rome III questionnaire (QPGS); parent report	0%
Játiva et al. (25)	Ecuador	General school population	8–15 years	417	Rome III questionnaire (QPGS Spanish version); self-report	0.7%
Helgeland et al. (26)	Norway	Pediatric outpatient clinic population	4–15 years	152	Rome III questionnaire (QPGS); parent report	2%
Van Tilburg et al. (27)	United States	Pediatric gastroenterology clinic population	4–18 years	135	Rome III questionnaire (QPGS); parent report and also self-report if 10 y.o. or older	3.5% by child report, 0.8% by parent report

While there are a relatively large number of general population studies, there are very few studies performed in a clinical setting, particularly in older children and adolescents. Malik and colleagues diagnosed rumination in 30 of 50 patients presenting to a pediatric gastroenterology for a primary complaint of vomiting (7). In two studies, older youth tended to report prevalence on the higher end when assessed in a pediatric gastroenterology clinic (3.5%; United States) or for a recurrent abdominal pain in a general outpatient clinic (2%; Norway) (26, 27). This gastroenterology clinic study also compared prevalence based on parent report as compared to self-report in youth 10 years of age or older and found a >4-fold higher prevalence by self-report (3.5 vs. 0.8%) (27). Utilizing the QPGS, we have previously shown a discordance in diagnosis between parent and child report across abdominal pain-associated functional gastrointestinal disorders (FGIDs); this may hold true for rumination, as well, but further evaluation is needed (28). While the prevalence of rumination has been studied in general populations, given the high variability from study-to-study, it is unclear whether the methodology is sufficient to identify all patients with rumination. More studies specifically assessing rumination in the general population are needed. Additionally, more studies are needed to understand the prevalence in a clinical setting, particularly in patients with other gastrointestinal symptoms given that rumination frequently is associated with other FGIDs (14, 23).

### Pathophysiology

It has been proposed that there may be three pathways to development of rumination: (1) a primary pathway involving premonitory urges (such as seen in tic disorders); (2) a pathway secondary to ongoing pathophysiology (such as gastroesophageal reflux); and, (3) a pathway secondary to psychosocial mechanisms stemming from a behavioral association which could begin in the presence of other FGIDs (e.g., to reduce pressure and pain in the stomach) and persist as a learned behavior in response to contextual clues (such as foods or changes in visceral sensation) after resolution of the other FGID (29). It is likely that there are overlapping mechanisms in individual patients.

Utilizing antroduodenal motility studies or high-resolution esophageal manometry (HREM) with esophageal impedance monitoring, the physiologic changes occurring during a rumination episode have been well-described and appear consistent irrespective of the triggering event. Although the intragastric pressure threshold has varied somewhat from study to study, rumination involves strong voluntary but generally unconscious contraction of the muscles of the abdominal wall promoting an increase in intragastric pressure with subsequent movement of gastric contents into the esophagus and mouth (i.e., the “R” or retrograde wave) (30, 31). Care needs to be taken to ensure that increases in abdominal pressure are not due to coughing or speaking. In controls, gastroesophageal reflux episodes are not associated with intragastric pressure increases of this magnitude (31). In youth with rumination, a significant portion of rumination episodes are preceded by gastroesophageal reflux (31, 32). Utilizing HREM and impedance, Rosen and colleagues described three distinct patterns in patients with primary rumination (i.e., where rumination is not induced by a reflux episode): lower esophageal sphincter (LES) relaxation without retrograde flow before the R wave in 51%, an R wave followed by LES relaxation in 20%, and an R wave without LES relaxation in 29% of episodes (32).

In the absence of gastroesophageal reflux as a triggering event, the etiology of rumination is not well-delineated, particularly in children. Rumination is frequently associated with other gastrointestinal symptoms or functional gastrointestinal disorders. Concomitant abdominal pain has been reported in 23–38%, nausea in 17–30%, and constipation in 21–23% of youth previously diagnosed with rumination (5, 7). This might suggest some shared pathophysiology or that other gastrointestinal conditions may predispose to development of rumination. Dysmotility, poor accommodation, and visceral hyperalgesia have been described in functional dyspepsia (FD), another FGID originating from the upper gastrointestinal tract (33). Gastric emptying has been evaluated in youth with rumination and delayed emptying reported in 30–46% (5, 8). Whether abnormal studies are indicative of poor emptying or an artifact created by frequent regurgitation is not clear. We found no studies assessing accommodation or visceral sensitivity in youth with rumination. In adults, impaired accommodation has been reported in <10% (34). In an adult study utilizing a barostat to distend the gastric fundus, patients with rumination reported increased nausea and bloating with no difference in gastric compliance or accommodation; however, the patients demonstrating no accommodation reported increased pain at increased pressures (35). Rumination patients also had increased gastric sensitivity during gastric distension (35). This may suggest a role for accommodation and/or visceral hypersensitivity in at least a subset of youth with rumination.

There is a paucity of studies evaluating inflammation in rumination syndrome. Gastrointestinal inflammation has been implicated in other functional gastrointestinal disorders, including functional dyspepsia, in youth (36). Specifically, associations have been demonstrated with increased density and activation of eosinophils and mast cells (36). Adult rumination syndrome has been associated with an increase in duodenal eosinophils and intraepithelial lymphocytes (37). Recently, we found an increase in gastric antral eosinophils and mast cells, as well as duodenal eosinophils and intraepithelial lymphocytes, in youth with rumination syndrome (38).

### Treatment

Diaphragmatic breathing has emerged as the mainstay of treatment. While open trials and randomized controlled trials of diaphragmatic breathing have contained some older adolescents, they are primarily studies which have been performed in adults. A systematic review of primarily adult studies assessing rumination, concluded that the efficacy of diaphragmatic breathing was supported by four chart reviews, two open clinical trials (*N*= 28 and *N*= 10), and one randomized trial (*N* = 23) which all demonstrated a significant decrease in daily regurgitation (29). It is believed that the primary mode of action of diaphragmatic breathing is that it creates a competing response to the abdominal wall contractions that create a rumination episode (39, 40). In adults, diaphragmatic breathing counteracts the increase in intragastric pressure and also increases pressure at the esophageal-gastric junction (41). While diaphragmatic breathing results in a significant decrease in rumination episodes, for many or most patients, rumination continues albeit at a lower rate (39, 40, 42). Other techniques for creating competing responses (e.g., chewing or sucking on hard candy or peppermints) have been reported in individual patients but not studied in controlled trials or in comparison to diaphragmatic breathing (29).

To the extent that stress is an exacerbating factor in rumination, diaphragmatic breathing also may provide indirect benefit by inducing a relaxation response that can further minimize the likelihood of rumination (43). Other commonly employed methods for addressing anxiety, such as cognitive behavioral therapy or hypnosis, are frequently utilized as part of multiple interventions in the treatment of rumination but have not been evaluated individually in controlled trials for the treatment of rumination.

As noted earlier, in a prospective study of patients in a pediatric gastroenterology clinic, Malik and colleagues evaluated 50 children presenting with vomiting and diagnosed rumination syndrome in 30 (7). Seven of these self-resolved without treatment after the initial consultation, counseling, and evaluation and 23 underwent education regarding the pathophysiology of rumination and were taught diaphragmatic breathing. Of these 23 patients, rumination resolved in 82.6% initially, although 28.5% of these relapsed (7). In a retrospective study of 52 children utilizing instruction in diaphragmatic breathing, biofeedback, relaxation training, and/or cognitive behavioral therapy, rumination resolved in 29.6%, improved in 55.6%, and was unchanged in 12.9% (5). In a group of 12 patients treated as inpatients with antidepressants, anxiolytics, and cognitive behavioral therapy with biofeedback or relaxation techniques, resolution or improvement was seen in 10 of 12 patients (44). In a long-term (9.5–48.5 months) follow-up study of patients treated for rumination, 40% reported a rumination-free period of 2 weeks−6 months and 19% a remission-free period of >6 months (43). However, over 70% of those with prolonged remission periods had recurrence, most often associated with stress, illness, specific foods, or menstruation—triggers also common for other FGIDs (43).

There have been very few studies evaluating medications in the treatment of rumination syndrome and most involve medication in combination with other treatment modalities. While acid-reduction therapy or promotility agents are frequently instituted to treat presumed gastroesophageal reflux, clinic experience (in the absence of prospective, controlled trials) would indicate they are not effective in rumination (29). Certainly, if a patient with suspected gastroesophageal reflux is not responsive to acid-reduction therapy (although ideally earlier), rumination should be considered. In the only prospective placebo-controlled trial we identified, Pauwels and colleagues assessed the effects of baclofen in 20 patients, ages 18–61 years, with suspected rumination or supra-gastric belching (45). Baclofen resulted in a higher rate of subjective improvement (63 vs. 26%), increased lower esophageal sphincter pressure, decreased transient lower esophageal relaxation, and decreased reflux episodes (45). A case series also reported decreased rumination in 12 adults treated with baclofen (46). These findings suggest that baclofen may be helpful in reducing rumination, at least in part, by decreasing gastroesophageal reflux which may induce rumination. Baclofen has been reported to be helpful in pediatric gastroesophageal reflux as well and thus could be considered in the treatment of rumination in children and adolescents (47).

While some patients may experience resolution of rumination following a basic introduction of competing responses such as diaphragmatic breathing, a significant proportion will continue to have on-going symptoms or experience relapse. This is particularly true for those with co-morbid anxiety or depression and when experiencing stress (43, 48). Most patients will be better served by an interdisciplinary team consisting of a medical provider, a mental health provider, and often a dietician, particularly in the setting of weight loss. Treatment begins with confirming the diagnosis, providing reassurance, and educating patients on the pathophysiology of rumination syndrome, including factors which may exacerbate symptoms. The role of the medical provider is to assess and treat co-morbid conditions (e.g., gastroesophageal reflux, constipation, nausea, abdominal pain) and/or biologic factors (e.g., inflammation, nerve sensitivity, motility issues) that may underlie or complicate rumination, and to consider medications to augment behavioral interventions for rumination. The role of the mental health provider is to assess and treat co-morbid psychological conditions that may serve a contributory role (e.g., anxiety, depression), improve autonomic balance and rest/digest functions via relaxation training, and engage the patient in habit reversal training focused on identification of effective competing responses and the optimal timing/triggers for their use.

## Conclusions and Future Directions

Pediatric rumination syndrome remains greatly understudied with regard to prevalence, pathophysiology, and particularly regarding treatment. There is a need to better define prevalence in both the primary care and subspecialty clinical settings, especially in patients presenting with vomiting or apparent gastroesophageal reflux. The history in these patients should include assessment of symptoms indicative of rumination. In patients where rumination is preceded by lower esophageal sphincter relaxation and/or reflux, there is a need for follow-up studies to determine whether treatment of gastroesophageal reflux results in resolution of rumination. Given that rumination is often accompanied by other gastrointestinal symptoms, or other FGIDs, and may be a compensatory mechanism learned to control discomfort, there is a need to determine whether treatment of co-morbid conditions results in improvement of rumination. If the triggering event is still present (whether stress, inflammation, gastroesophageal reflux, etc.), it may be more difficult to effectively treat rumination and harder to gain adherence to future repeat behavioral treatment efforts following treatment failure. Although diaphragmatic breathing likely has benefit for rumination syndrome, significant long-term efficacy in youth has yet to be demonstrated with relatively high rates of relapse and short duration of symptom-free periods. It needs to be studied and compared to other competing responses (e.g., gum chewing or sucking on hard candy, particularly peppermints as these may have additional benefits in relaxing smooth muscle and improving accommodation). As most pediatric reports have included multiple treatment modalities, individual components need to be studied to understand which are effective (alone or in combination) to increase the efficiency of care.

## Author Contributions

All authors contributed to conceptualization of this review. MM, SR, and HF completed the initial literature review. MM, SR, HF, and CF completed the initial manuscript draft. All authors provided critical review and editing.

## Conflict of Interest

The authors declare that the research was conducted in the absence of any commercial or financial relationships that could be construed as a potential conflict of interest.

## Publisher's Note

All claims expressed in this article are solely those of the authors and do not necessarily represent those of their affiliated organizations, or those of the publisher, the editors and the reviewers. Any product that may be evaluated in this article, or claim that may be made by its manufacturer, is not guaranteed or endorsed by the publisher.

## References

[1] Hyams JS Di Lorenzo C Saps M Shulman RJ Staiano A van Tilburg M. Childhood functional gastrointestinal disorders: child/adolescent. Gastroenterology. (2016) 150:1456–68. 10.1053/j.gastro.2016.02.015 16678566 PMC7104693

[2] Olden KW. Rumination. Curr Treat Options Gastroenterol. (2001) 4:351–8. 10.1007/s11938-001-0061-z 11469994

[3] Benninga MA Nurko S Faure C Hyman PE St. James Roberts I Schechter NL. Childhood functional gastrointestinal disorders: child/toddler. Gastroenterology. (2016) 150:1443–55. 10.1053/j.gastro.202.02.016 27144631

[4] O'Brien MD Bruce BK Camilleri M. The rumination syndrome: clinical features rather than manometric diagnosis. Gastroenterology. (1995) 108:1024–9. 10.1016/0016-5085(95)90199-x 7698568

[5] Chial HJ Camilleri M Williams DE Litzinger K Perrault J. Rumination syndrome in children and adolescents: diagnosis, treatment, and prognosis. Pediatrics. (2003) 111:158–62. 10.1542/peds.111.1.158 12509570

[6] Nikaki K Rybak A Nakagawa K Rawat D Yazaki E Woodland P. Rumination syndrome in children presenting with refractory gastroesophageal symptoms. J Pediatr Gastroenterol Nutr. (2020) 70:330–5. 10.1097/MPG.0000000000002569 32079888

[7] Malik R Srivastava A Yachha SK Poddar U. Chronic vomiting in children: a prospective study reveals rumination syndrome is an important etiology that is under diagnosed and untreated. Indian J Gastroenterol. (2020) 39:196–203. 10.1007/s12664-020-01025-x 32436177

[8] Alioto A Di Lorenzo C Montgomery ML Yacob D. High cost and low yield: The diagnostic evaluation of rumination syndrome in pediatrics. J Pediatr. (2017) 185:155–9. 10.1016/j.jpeds.2017.02.009 28256211

[9] Monagas J Ritwik P Kolomensky A Acosta J Kay D Clendaniel L. Rumination syndrome and dental erosions in children. J Pediatr Gastroenterol Nutr. (2017) 64:930–2. 10.1097/MPG.0000000001395 27579694

[10] Scarpato E Kolacek S Jojkic-Pavkov D Konjik V Živković N Roman E. Prevalence of functional gastrointestinal disorders in children and adolescents in the Mediterranean region of Europe. Clin Gastroenterol Hepatol. (2018) 16:870–6. 10.1016/j.cgh.2017.11.005 29129667

[11] Robin SG Keller C Zwiener R Hyman PE Nurko S Saps M. Prevalence of pediatric functional gastrointestinal disorders utilizing the Rome IV criteria. J Pediatr. (2018) 195:134–9. 10.1016/j.jpeds.2017.12.012 29398057

[12] Steutel NF Zeevenhooven J Scarpato E Vandenplas Y Tabbers MM Staiano A. Prevalence of functional gastrointestinal disorders in European infants and toddlers. J Pediatr. (2020) 221:107–14. 10.1016/j.jpeds.2020.02.076 32446468

[13] Altamimi E Scarpato E Saleh I Tantawi K Alassaf M Ijam M. National prevalence of functional gastrointestinal disorders in Jordanian children. Clin Exp Gastroenterol. (2020) 13:267–72. 10.2147/CEG.S256276 32821146 PMC7423213

[14] Peralta-Palmezano JJ Guerrero-Lozano R. Prevalence of functional disorders in school children and adolescents. Korean J Gastroenterol. (2019) 73:207–12. 10.4166/kjg.2019.73.4.207 31030457 PMC12285841

[15] Bouzios I Chouliaras G Chrousos GP Roma E Gemou-Engesaeth V. Functional gastrointestinal disorders in Greek children based on Rome III criteria: identifying the child at risk. Neurogastroenterol Motil. (2017) 29:e12951. 10.1111/nmo.12951 27679978

[16] Murray HB Thomas JJ Hinz A Munsch S Hilbert A. Prevalence in primary school youth of pica and rumination behavior: the understudied feeding disorders. Int J Eat Disord. (2018) 51:994–8. 10.1002/eat.22898 30175409

[17] Chogle A Velasco-Benitez CA Koppen IJ Moreno JE Ramírez Hernández CR Saps M. A population-based study on the epidemiology of functional disorders in young children. J Pediatr. (2016) 179:139–43.e1. 10.1016/j.jpeds.2016.08.095 27726867

[18] Devanarayana NM Adhikari C Pannala W Rajindrajith S. Prevalence of functional gastrointestinal diseases in a cohort of Sri Lankan adolescents: comparison between Rome II and Rome III criteria. J Trop Pediatr. (2011) 57:34–9. 10.1093/tropej/fmq039 20525779

[19] Bhatia V Deswal S Seth S Kapoor A Sibal A Gopalan S. Prevalence of functional gastrointestinal disorders among adolescents in Delhi based on Rome III criteria: a school-based survey. Indian J Gastroenterol. (2016) 35:294–8. 10.1007/s12664-016-0680-x 27554498

[20] Hartmann AS Poulain T Vogel M Hiemisch A Kiess W Hilbert A. Prevalence of pica and rumination behaviors in German children aged 7-14 and their associations with feeding, eating, and general psychopathology: a population-based study. Eur Child Adolesc Psychiatry. (2018) 27:1499–508. 10.1007/s00787-018-1153-9 29675593

[21] Saps M Velasco-Benitez CA Langshaw AH Ramírez-Hernández CR. Prevalence of functional gastrointestinal disorders in children and adolescents: comparison between Rome III and Rome IV criteria. J Pediatr. (2018) 199:212–6. 10.1016/j.jpeds.2018.03.037 29747935

[22] Sagawa T Okamura S Kakizaki S Zhang Y Morita K Mori M. Functional gastrointestinal disorders in adolescents and quality of life. J Gastroenterol Hepatol. (2013) 28:285–90. 10.1111/j.1440-1746.2012.07257.x 22988951

[23] Rajindrajith S Devanarayana NM Crispus Perera BJ. Rumination syndrome in children and adolescents: a school survey assessing prevalence and symptomatology. BMC Gastroenterol. (2012) 12:163. 10.1186/1471-230X-12-163 23157670 PMC3538663

[24] Lewis ML Palsson OS Whitehead WE van Tilburg MAL. Prevalence of functional gastrointestinal disorders in children and adolescents. J Pediatr. (2016) 177:39–43.e3. 10.1016/j.jpeds.2016.04.008 27156185

[25] Játiva E Velasco-Benítez CA Koppen IJN Játiva-Cabezas Z Saps M. Prevalence of functional gastrointestinal disorders in schoolchildren in Ecuador. J Pediatr Gastroenterol Nutr. (2016) 63:25–8. 10.1097/MPG.0000000000001108 26771768

[26] Helgeland H Flagstad G Grøtta J Vandvik PO Kristensen H Markestad T. Diagnosing pediatric functional abdominal pain in children (4-15 years old) according to Rome III criteria: results from a Norwegian prospective study. J Pediatr Gastroenterol Hepatol. (2009) 49:309–15. 10.1097/MPG.0b013e31818de3ab 19525874

[27] van Tilburg MA Squires M Blois-Martin N Leiby A Langseder A. Test of the child/adolescent Rome III criteria: agreement with physician diagnosis and daily symptoms. Neurogastroenterol Motil. (2013) 25:302–e246. 10.1111/nmo.12056 23216900

[28] Schurman JV Friesen CA Danda CE Andre A Welchert E Lavenbarg T. Diagnosing functional abdominal pain with the Rome criteria: parent, child, and clinician agreement. J Pediatr Gastroenterol Nutr. (2005) 41:291–5. 10.1097/01.mpg.0000178438.64675.c4 16131983

[29] Murray HB Juarascio AS Di Lorenzo C Drossman DA Thomas JJ. Diagnosis and treatment of rumination syndrome: a critical review. Am J Gastroenterol. (2019) 114:562–78. 10.14309/ajg.0000000000000060 30789419 PMC6492032

[30] Singendonk MMJ Oors JM Bredenoord AJ Omari TI van der Pol RJ Smits MJ. Objectively diagnosing rumination syndrome in children using esophageal pH-impedance and manometry. Neurogastroenterol Motil. (2017) 29:e12996. 10.1111/nmo.12996 28078818

[31] Grunder FR Aspirot A Faure C. High-resolution esophageal manometry patterns in children and adolescents with rumination syndrome. J Pediatr Gastroenterol Nutr. (2017) 65:627–32. 10.1097/MPG.0000000000001618 29072581

[32] Rosen R Rodriguez L Nurko S. Pediatric rumination subtypes: a study using high-resolution esophageal manometry with impedance. Neurogastroenterol Motil. (2017) 29:e12998. 10.1111/nmo.12998 28002887 PMC5393952

[33] Rosen JM Cocjin JT Schurman JV Colombo JM Friesen CA. Visceral hypersensitivity and electromechanical dysfunction as therapeutic targets in pediatric functional dyspepsia. World J Gastrointest Pharmacol Ther. (2014) 5:122–38. 10.4292/wjgpt.v5.i3.122 25133041 PMC4133438

[34] Bredenoord AJ Chial HJ Camilleri M Mullan BP Murray JA. Gastric accommodation and emptying in evaluation of patients with upper gastrointestinal symptoms. Clin Gastroenterol Hepatol. (2003) 1:264–72. 15017667

[35] Thumshirn M Camilleri M Hanson RB Williams DE Schei AJ Kammer PP. Gastric mechanosensory and lower esophageal sphincter function in rumination syndrome. Am J Physiol. (1998) 275:G314–21. 10.1152/ajpgi.1998.275.2.G314 9688659

[36] Friesen CA Schurman JV Colombo JM Abdel-Rahman SM. Eosinophils and mast cells as therapeutic targets in pediatric functional dyspepsia. World J Gastrointest Pharmacol Ther. (2013) 4:86–96. 10.4292/wjgpt.v4.i4.86 24199024 PMC3817289

[37] Halland M Talley NJ Jones M Murray JA Cameron R Walker MM. Duodenal pathology in patients with rumination syndrome: duodenal eosinophilia and increased intraepithelial lymphocytes. Dig Dis Sci. (2019) 64:832–7. 10.1007/s10620-018-5387-7 30478768

[38] Friesen HJ Rosen J Low Kapalu C Singh M Spaeth T Cocjin JT. Mucosal eosinophils, mast cells, and intraepithelial lymphocytes in youth with rumination syndrome. Neurogastroenterol Motil. (2021) e14155. 10.1111/nm0.14155 33837997

[39] Barba E Burri E Accarino A Malagelada C Rodriguez-Urrutia A Soldevilla A. Biofeedback-guided control of abdominothoracic muscular activity reduces regurgitation episodes in patients with rumination. Clin Gastroenterol Hepatol. (2015) 13:100–6.e1. 10.1016/j.cgh.2014.04.018 24768808

[40] Barba E Accarino A Soldevilla A Malagelada JR Azpiroz F. Randomized, placebo-controlled trial of biofeedback for the treatment of rumination. Am J Gastroenterol. (2016) 111:1007–13. 10.1038/ajg.2016.197 27185077

[41] Halland M Parthasarathy G Bharucha AE Katzka DA. Diaphragmatic breathing for rumination syndrome: efficacy and mechanisms of action. Neurogastroenterol Motil. (2016) 28:384–91. 10.1111/nmo.12737 26661735

[42] Tucker E Knowles K Wright J Fox MR. Rumination variations: aetiology and classification of abnormal behavioural responses to digestive symptoms based on high-resolution manometry studies. Aliment Pharmacol Ther. (2013) 37:263–74. 10.1111/apt.12148 23173868

[43] Alioto A Di Lorenzo C. Long-term follow-up of adolescents treated for rumination in an inpatient setting. J Pediatr Gastroenterol Nutr. (2018) 66:21–5. 10.1097/MPG.0000000000001632 28505048

[44] Khan S Hyman PE Cocjin J Di Lorenzo C. Rumination syndrome in adolescents. J Pediatr. (2000) 136:528–31. 10.1016/s0022-3476(00)90018-0 10753253

[45] Pauwels A Broers C Van Houtte B Rommel N Vanuytsel T Tack J. A randomized double-blind, placebo-controlled, cross-over study using baclofen in the treatment of rumination syndrome. Am J Gastroenterol. (2018) 113:97–104. 10.1038/ajg.2017.441 29206813

[46] Blondeau K Boecxstaens V Rommel N Farré R Depeyper S Holvoet L. Baclofen improves symptoms and reduces postprandial flow events in patients with rumination and supragastric belching. Clin Gastroenterol Hepatol. (2012) 10:379–84. 10.1016/j.cgh.2011.10.042 22079512

[47] Vadlamudi NB Hitch MC Dimmitt RA Thame KA. Baclofen for the treatment of pediatric GERD. J Pediatr Gastroenterol Nutr. (2013) 57:808–12. 10.1097/MPG.0b013e3182a2747b 23838820

[48] Alioto A Yacob D Yardley HL Di Lorenzo C. Inpatient treatment of rumination syndrome: outcomes and lessons learned. Clin Pract Pediatr Psychol. (2015) 3:304–13. 10.1037/cppp0000115

